# Passive transfer of collagen XVII-specific antibodies induces sustained blistering disease in adult mice

**DOI:** 10.1186/1750-1172-8-17

**Published:** 2013-01-29

**Authors:** Mircea Teodor Chiriac, Emilia Licarete, Alexandra Gabriela Sas, Andreea Maria Rados, Iulia Lupan, Anca Mirela Chiriac, Hilda Speth, Vlad Pop-Vancia, Iacob Domsa, Alina Sesarman, Octavian Popescu, Cassian Sitaru

**Affiliations:** 1 Department of Biology, Babes-Bolyai University, Cluj-Napoca, Romania; 2 Molecular Biology Center, Interdisciplinary Research Institute on Bio-Nano-Sciences, Babes-Bolyai University, Cluj-Napoca, Romania; 3 Medical Clinic III, University of Medicine and Pharmacy, Cluj-Napoca, Romania; 4 Medical Clinic IV, University of Medicine and Pharmacy, Cluj-Napoca, Romania; 5 Centre for Biological Signalling Studies (BIOSS), University of Freiburg, Freiburg, Germany; 6 Institute of Biology, Romanian Academy, Bucharest, Romania; 7 Department of Dermatology, University of Freiburg, Freiburg, Germany

**Keywords:** Autoimmunity, Collagen XVII, Inflammation, Skin

## Abstract

**Background:**

Bullous pemphigoid is a subepidermal blistering disorder associated with tissue-bound and circulating autoantibodies directed mainly to the hemidesmosomal component collagen XVII. While recapitulating the main immunopathological features of the human disease, frank skin blistering does not develop in the absence of skin rubbing in experimental pemphigoid models that have been established in neonatal mice. Moreover, due to their experimental design they only allow for short-term disease observation. In the present study we aimed to establish a model that reproduces the frank skin blistering seen in patients and allows for longer observation times.

**Methods:**

Rabbit and sheep antibodies specific to several fragments of collagen XVII were generated and the purified antibodies were passively transferred into adult mice.

**Results:**

Collagen XVII-specific IgG bound to the basal membrane of the skin and mucous membranes activating murine complement *in vivo*. Mice injected with collagen XVII-specific antibodies, in contrast to mice receiving control antibodies, developed frank skin blistering disease, reproducing human bullous pemphigoid at the clinical, histological and immunopathological levels. Titres of circulating IgG in the serum of mice correlated with the extent of the clinical disease. Mice receiving sheep antibodies specific to murine collagen XVII showed an early onset and a more active disease when compared to litter mates receiving specific rabbit antibodies.

**Conclusion:**

This novel animal model for bullous pemphigoid should facilitate further investigations of the pathogenesis of bullous pemphigoid and the development of innovative therapies for this disease.

## Background

Bullous pemphigoid (BP) is the most common autoimmune blistering disease in Western Europe and USA [1-4]. Immunopathologically, the disease is characterized by the presence of tissue-bound and circulating autoantibodies directed against the dermal-epidermal junction (DEJ). Antibodies in BP patients mainly recognize BP180 (bullous pemphigoid antigen of 180kDa) also known as collagen (C)XVII, a transmembrane protein with a type II orientation that spans the lamina lucida, and to a lesser extent BP230 (bullous pemphigoid antigen of 230kDa), an intracellular hemidesmosomal protein [5-10]. Since these proteins contribute to the tight anchoring of basal keratinocytes on the underlying basal membrane, disruption of their function either through genetic defects or immunopathological mechanisms results in a dermal-epidermal separation that ultra-structurally localizes within the lamina lucida [10-15].

The pathogenic relevance of autoantibodies in BP is supported by compelling evidence: 1) BP autoantibodies were shown to recruit and activate leukocytes resulting in dermal-epidermal separation in cryosections of human skin [16]; 2) Antibodies against mouse [17] and human [14] BP180/ CXVII induce subepidermal blisters when passively transferred into wild-type or CXVII-humanized mice, respectively; in addition, antibodies to human type XVII collagen cross the placenta of immunized mice and induce disease in type XVII collagen-humanized neonates [18]; 3) Transfer of splenocytes from mice immunized against human type XVII collagen into Rag2^−/−^/ CXVII-humanized mice results in sustained immunoglobulin G (IgG) production and BP phenotype [19]; 4) Autoantibodies in the majority of BP patients recognize the non-collagenous 16^th^ (NC16)A domain of BP180/ CXVII and pre-adsorption of pathogenic sera with NC16A abolished their pathogenic potential both *in vivo*[15,18] and *ex vivo*[16].

Despite important advances in disease management over the last decades, BP is still a disease with one-year mortality rates of up to 40% [20]. The existence of animal models reproducing the disease is essential for our understanding of pathomechanisms with major implications for afflicted individuals. The most direct approach for reproducing autoantibody-induced autoimmune diseases *in vivo* has been to inject patients’ serum or purified antibodies specific to culprit autoantigens into healthy individuals. The method pioneered in the 1950s by the Harrington–Hollingsworth Experiment wherein Dr. Harrington received blood from an idiopathic thrombocytopenic purpura patient was first used to test the hypothesis that platelet destruction was caused by a factor circulating in the patient’s blood [21,22]. Future studies using the passive transfer of IgG into laboratory animals demonstrated the pathogenic effect of antibodies in several diseases, including myasthenia gravis [23], pemphigus vulgaris [24] and pemphigus foliaceus [25]. Previous attempts to reproduce BP by this “classical” transfer of disease through antibodies from patients into experimental animals were unsuccessful [26-30]. The failure to transfer the disease in mice has been explained by a lack of reactivity of patients’ autoantibodies with the murine BP180/ CXVII-specific due to the low degree of homology between the human and mouse type XVII collagen [14,15,17,18,31]. A further reason for the lack of pathogenicity of pemphigoid patient’s autoantibodies in mice is related to their significantly weaker capacity of activating mouse innate immune factors when compared to human complement and granulocytes [32]. The “alternative” strategy of generating antibodies to the murine form of type XVII collagen by immunizing rabbits and then transferring rabbit antibodies into mice [17] has been used successfully for developing *in vivo* models for several other autoimmune diseases such as pemphigus vulgaris [33], anti-epiligrin cicatricial pemphigoid [34], and epidermolysis bullosa acquisita [35]. Among these, the neonatal BP models have some major shortcomings including the fact that frank skin blistering does not occur and the very short observation times that precludes adequately dissecting disease pathogenesis and developing therapeutic strategies. In the present study, we aimed at addressing these shortcomings by developing a novel passive transfer model for bullous pemphigoid in adult mice. We generated antibodies against the murine BP180/ CXVII by immunizing rabbit and sheep with recombinant forms of the murine antigen. After being passively injected into adult wild type mice, these antibodies bound to the DEJ, activated complement and recruited inflammatory cells resulting in tissue damage. The phenotype of the disease mimicked human BP at the clinical, immunological and histopathological levels. Titres of BP180/ CXVII-specific antibodies in the peripheral blood of injected animals correlated well with disease activity. Immune sheep sera showed higher BP180/ CXVII-specific levels compared to rabbit antibodies and induced more extensive disease after their passive transfer in mice. This model provides a solid basis for further pathogenetic studies in BP and for the development of new therapeutic approaches.

## Materials and methods

### Mice

Six- to eight-week-old BALB/c mice with a body weight of approximately 20 g were used. Mice were obtained from the Cantacuzino Institute (Bucharest, Romania) and housed at our animal facility. All injections and bleedings were performed on mice narcotized by administration of a mixture of ketamine (100 μg/g) and xylazine (15 μg/g). Mice received subcutaneously 10 mg of ammonium sulfate-precipitated BP180/ CXVII-specific antibodies from either rabbit (end-titre 12.800-25.600) or sheep (end-titre 102.400) every second day for two weeks. Control mice received the same amounts of normal preimmune rabbit or sheep antibodies, referred to hereafter as control antibodies. The experiments were approved by the Ethics Committee (Babes-Bolyai University Cluj-Napoca no. 1146/2009 and 31458/2010) and performed by qualified personnel.

### Heterologous expression of murine BP180/ CXVII fragments

Three extracellular and one intracellular fragments of murine BP180/ CXVII were expressed as glutathione-*S*-transferase (GST) fusion proteins as described [32]. These were designated GST-mCXVII-EC1, GST-mCXVII-EC3, GST-mCXVII-EC7 and GST-mCXVII-IC2, and contain murine collagen XVII sequences stretching from amino acid positions 498–580, 856–901, 1030–1134, and 186–475, respectively. The proteins were produced using the recombinant vectors pGEX-mCOL17-EC1, pGEX-mCOL17-EC3, pGEX-mCOL17-EC7 and pGEX-mCOL17-IC2 in *Escherichia coli* and purified by glutathione agarose chromatography [32].

### Generation of BP180/ CXVII-specific rabbit and sheep antibodies

Three New Zealand White rabbits and one sheep were immunized subcutaneously with either 200 μg or 400 μg, respectively, of a mixture of the four purified recombinant fragments of murine type XVII collagen (GST-mCXVII-EC1, GST-mCXVII-EC3, GST-mCXVII-EC7 and GST-mCXVII-IC2 in a molar ratio of 2:1:1:1) mixed with Freund’s complete adjuvant. The animals were boosted twice with the same protein preparation in incomplete Freund’s adjuvant at two weeks intervals. Control antibodies were collected before the first immunization and immune sera were obtained at regular intervals and characterized by immunofluorescence (IF) microscopy on cryosections of murine skin. For the passive transfer experiments, sera from rabbits were pooled and immunoglobulins were isolated by ammonium sulfate precipitation. Sheep antibodies were also subjected to ammonium sulfate precipitation before passive transfer experiments. Protein concentration was measured spectrophotometrically at 280 nm [16].

### Characterization of murine BP180/ CXVII-specific antibodies

Frozen skin sections were prepared from tissue biopsies of mice and antibodies against murine BP180/ CXVII were analyzed by IF microscopy using 100-fold diluted antibodies specific to rabbit (DakoCytomation) or sheep IgG (Abcam). Complement-fixing activity of antibodies to the DEJ was determined as previously described [35]. Recombinant proteins were fractionated by 12.5% and 5% SDS-PAGE, transferred to nitrocellulose, and analyzed by immunoblotting [16] using HRP-conjugated secondary antibodies (DakoCytomation) and 3,3’ diaminobenzidine (Merck) as a chromogenic substrate.

### Induction of disease *in vivo* and phenotype assessment

Mice were examined daily for their general condition and for evidence of lesions (i.e., erythema, blisters, erosions, crusts and alopecia). The extent of skin disease (disease activity) was scored as follows: 0, no lesions; 1, < 10 lesions or < 1% of the skin surface; 2, > 10 lesions or 1-5% of the skin surface; 3, 5-10%; 4, 10-20%; and 5, > 20% involvement of the skin surface [36]. To evaluate the correlation of antibody titres with the extent of disease, sera were obtained from mice at three different time points (day 0, day 7 and day 14) and assayed by indirect IF microscopy on frozen sections of murine skin [37]. Skin biopsies of perilesional and lesional skin and mucous membranes were obtained at the end of the observation period and assayed for immuno-reactants deposition by direct IF and hematoxylin and eosin staining, respectively. The staining intensity of immunoreactants in the skin of injected mice was assessed semi-quantitatively using a score comprising 0, for no staining; 1, focal, faint staining; 2, faint staining, 3, medium; and 4, intense staining [36].

### Statistical analysis

To estimate the correlation between titres of circulating antibodies as detected by indirect IF microscopy, and disease activity, Spearman’s rank correlation test was applied. Differences in clinical disease activity among groups were calculated using the Chi-square test. All means are presented ± s.e.m.

## Results

### Generation and characterization of BP180/ CXVII-specific antibodies

**Figure 1:**
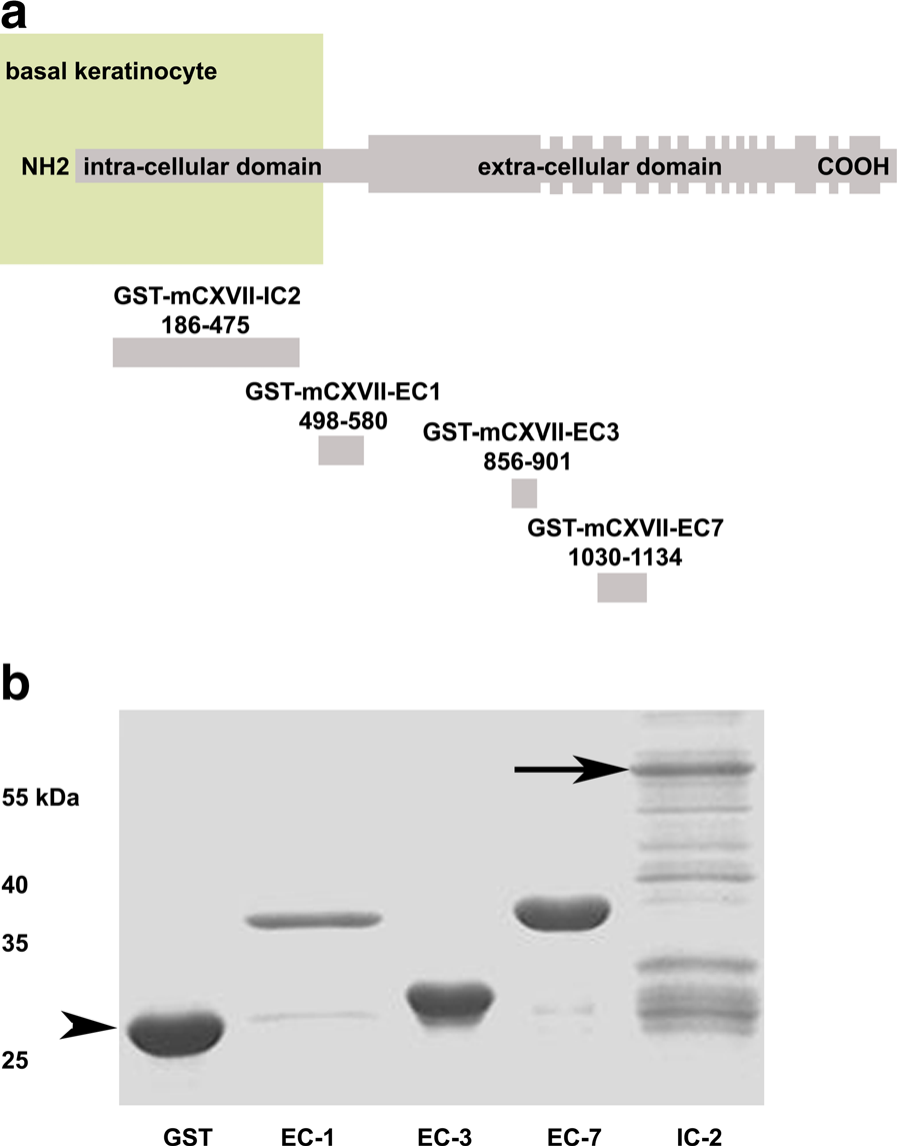
**Recombinant fragments of murine BP180/ CXVII.** (**a**) BP180/ CXVII is composed of an intracellular N-terminal fragment localized to the hemidesmosomal plaque of basal keratinocytes with a long extracellular portion containing 15 collagenous domains (filled rectangles) separated by non-collagenous regions protruding into the basal membrane. Four fragments of murine type XVII collagen cDNA cloned in pGEX-6P-1 were expressed in *E. coli*. Amino acid positions are shown below each fragment (the diagram is not at scale). (**b**) When run on an SDS-PAGE gel the proteins migrated correspondingly to their calculated molecular weights of 37, 32, 39 and 57 kDa, respectively. In lanes 1 through 6 we loaded: molecular weight marker, GST, GST-mCXVII-EC1, GST-mCXVII-EC3, GST-mCXVII-EC7 and GST-mCXVII-IC2. The arrow indicates GST-mCXVII-IC2 at 57kDa and the arrowhead indicates GST at 27kDa.

**Figure 2:**
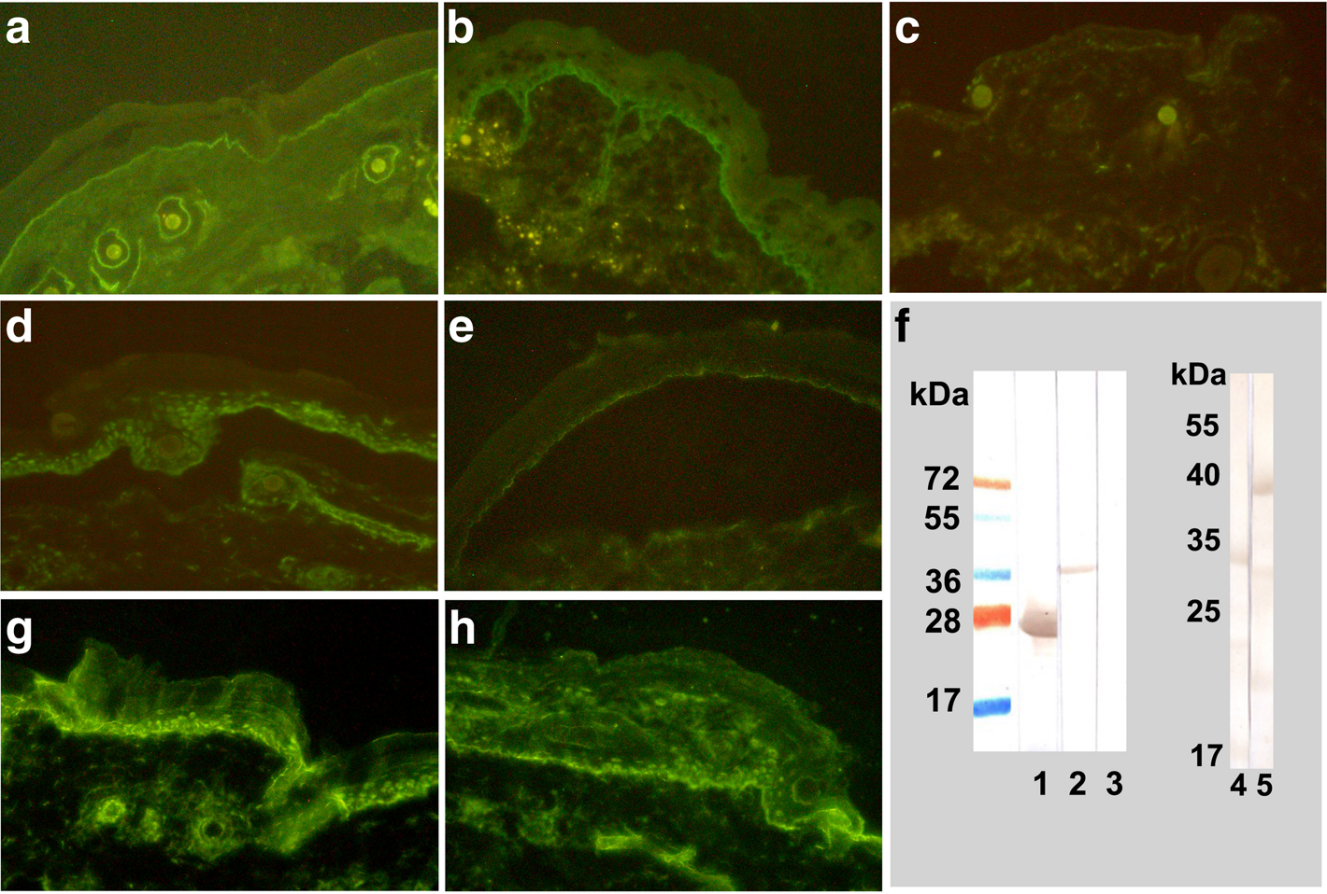
**Antibodies from immunized animals recognize mouse BP180/ CXVII and activate complement*ex vivo*.** Serum was obtained from immunized animals at different time points. Immune rabbit (**a**) and sheep (**b**) antibodies bound to the basal membrane of the mouse skin. In contrast, antibodies obtained from a rabbit before the first immunization (**c**) did not recognize the antigen *in situ*. When incubated with mouse split skin, IgG from the immune sera of both the rabbit (**d**) and sheep (**e**) bound to the epidermal side of the 1 NaCl-split skin (magnifications 200x). (**f**) Pathogenic antibodies in contrast to control antibodies recognized recombinant forms of BP180/ CXVII by immunoblotting; in the left hand panel: 1, GST and 2, GST-mCXVII-EC1 incubated with immune rabbit serum; 3, GST-mCXVII-EC1 incubated with control rabbit antibodies as a negative control; in the right hand panel: 4, GST-mCXVII-EC3 and 5, GST-mCXVII-EC7 incubated with immune rabbit serum. BP180/ CXVII-specific antibodies from the rabbit (**g**) and sheep (**h**) activated complement when incubated on cryosections of murine skin (magnifications 200x).

Four fragments of murine BP180/ CXVII cloned in a prokaryotic expression vector were expressed in *E. coli* (Figure 1a). The proteins, purified by glutathione-affinity chromatography, migrated consistently with their calculated masses of 37, 32, 39 and 57 kDa when separated by SDS-PAGE (Figure 1b). Circulating antibodies obtained from animals immunized with recombinant fragments of murine BP180/ CXVII were tested for their ability to recognize the protein *in situ* and by immunoblotting. Antibodies from both rabbit and sheep showed a linear staining of the basal membrane by IF microscopy using murine skin as a substrate (Figures 2a, 2b and Table 1). In contrast, antibodies obtained before the first immunization did not bind to the DEJ (Figure 2c). When incubated with 1M NaCl–split mouse skin, antibodies from immunized rabbits (Figure 2d) and sheep (Figure 2e) stained the epidermal side of the substrate. Additionally, sera from immune rabbits (Figure 2g) and sheep (Figure 2h), but not control antibodies (not shown) elicited deposition of murine C3 at the DEJ, as shown by the complement fixation test using murine skin as a substrate. By immunoblot analysis, antibodies from immune sera, in contrast to control sera, recognized recombinant forms of BP180/ CXVII (Figure 2f).

**Table 1:** Antibodies to the dermal-epidermal junction (DEJ) produced for this study

**Serum**	**Species**	**Molecular target**^**a**^	**IF**^**b**^	**CBT**^**c**^
R1-mCXVII	Rabbit	BP180/ CXVII	2560	+
R2-mCXVII	Rabbit	BP180/ CXVII	640	+
R3-mCXVII	Rabbit	BP180/ CXVII	1280	+
S1-mCXVII	Sheep	BP180/ CXVII	20480	+

^**a**^ antibodies were elicited against a mixture of BP180/ CXVII fragments (see methods).

^**b**^ titre of indirect immunofluorescence (IF) microscopy on mouse skin sections.

^**c**^ C3 deposition at the DEJ of mouse skin by the complement binding test (CBT).

### BP180/ CXVII-specific antibodies induce frank skin blistering when passively transferred into adult mice

**Figure 3:**
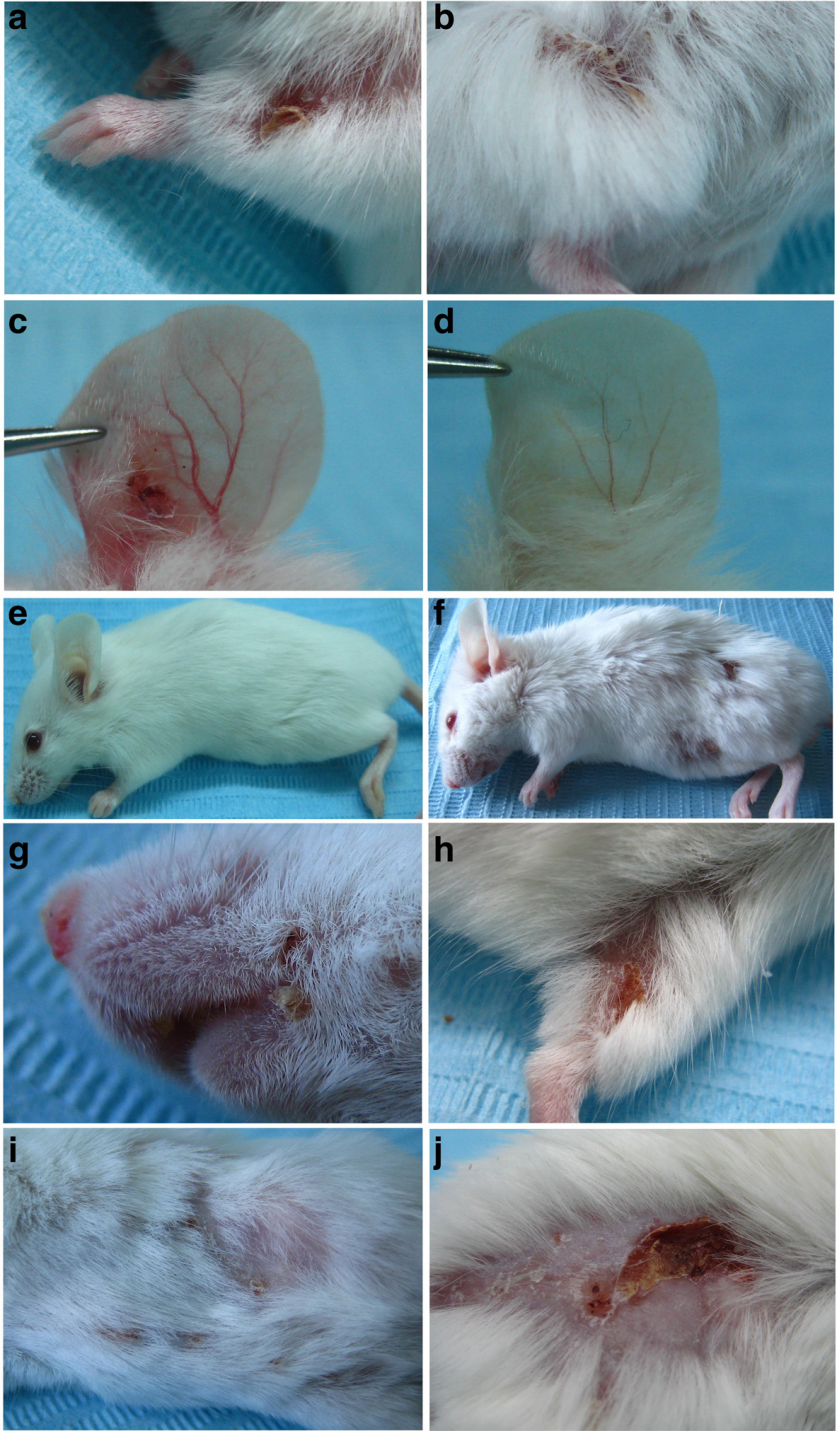
**BP180/ CXVII-specific antibodies induce blistering skin disease when passively transferred into adult mice.** Mice receiving rabbit antibodies (n=10) to murine BP180/ CXVII developed skin lesions at different sites including the (**a**) front limbs, the (**b**) hind limbs and the (**c**) ears. In contrast, the ear of a mouse receiving control rabbit antibodies remained unaffected (**d**) as did a mouse receiving control sheep antibodies (**e**). Mice injected with (**f**) pathogenic sheep antibodies (n=10) presented an even more extensive and generalized disease. Lesions including erosions partly covered by crusts were recorded on the (**g**) snout, and (**h**) hind limb of a mouse receiving sheep antibodies to murine BP180/ CXVII. More advanced disease was characterized by blisters, erosions and alopecia affecting both the (**i**) abdomen and the (**j**) back of mice injected with sheep BP180/ CXVII-specific IgG.

The passive transfer of rabbit BP180/ CXVII-specific IgG resulted in spontaneous blistering even in the absence of skin rubbing or other investigator manipulations in adult mice (Figure 3a-c). In mice (n=10) receiving 10 mg/injection of BP180/ CXVII-specific rabbit IgG lesions first appeared 9–10 days after the primary injection. In these mice, initial blisters evolved into erosions partly covered by crusts on an erythematous background. Mice receiving an equivalent amount of control rabbit (n=6) or sheep antibodies (n=6) showed no lesions at any time-point during the observation period (Figure 3d and e, respectively). To test whether antibodies produced in other species can transfer the disease, we injected adult mice (Figure 3f) with 10 mg/injection of sheep BP180/ CXVII-specific antibodies every second day (n=10). Initial lesions, including blisters and erosions appeared after 5–6 days in mice injected with these sheep antibodies. Lesions developed at different sites, including the ears, the snout (Figure 3g), hind limbs (Figure 3h), abdomen (Figure 3i) and the back (Figure 3j). In general, more extensive disease developed around the injection sites, but few lesions also developed at distant sites.

### Mice injected with BP180/ CXVII-specific antibodies showed immunopathological features of human BP

**Figure 4:**
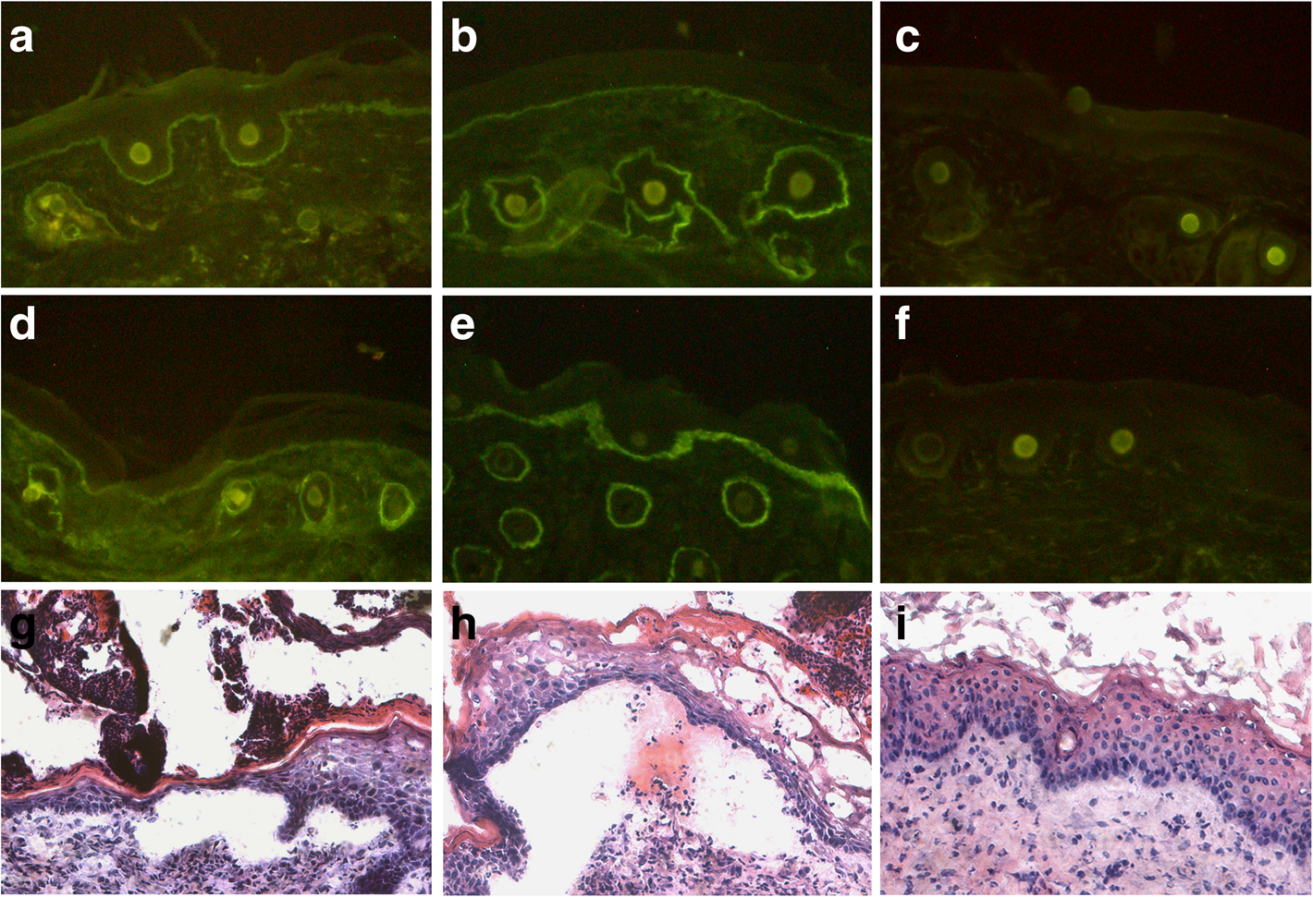
**Diseased mice show immuno- and histo-pathological features of the pemphigoid disease.** At the end of the observation period, mice were sacrificed and perilesional biopsies were analyzed for immunoreactants deposition. By IF microscopy, the perilesional skin of a diseased mouse injected with (**a**) rabbit or (**b**) sheep pathogenic antibodies showed linear deposition of murine IgG at the DEJ. Additionally, tissue bound (**d**) rabbit and (**e**) sheep antibodies were shown to activate murine C3 *in vivo* as revealed by direct IF microscopy. In contrast, no (**c**) IgG or (**f**) complement deposits were found in mice injected with control sheep antibodies. The lesional skin of a mouse receiving (**g**) rabbit or (**h**) sheep BP180/ CXVII-specific IgG shows a dermal-epidermal separation accompanied by an inflammatory infiltrate. In contrast, the skin of a mouse receiving (**i**) control sheep antibodies showed no histopathological signs of disease (magnifications, 200x).

At the end of the observation period, mice receiving pathogenic antibodies and control mice were sacrificed and biopsies of perilesional skin and oesophagus were analyzed for immunoreactant deposition. In mice with clinical signs of disease, we identified linear deposition of rabbit (Figure 4a) and sheep (Figure 4b) IgG along the basement membrane of both the skin and oesophagus (not shown). In addition, deposits of complement C3 were detected at the DEJ of mice injected with rabbit (Figure 4d) and sheep (Figure 4e) BP180/ CXVII-specific IgG. In contrast, mice receiving control rabbit (not shown) or sheep antibodies did not show deposition of IgG (Figure 4c) or mouse C3 (Figure 4f) in the perilesional skin or oesophagus (not shown). Mouse IgG could be detected in the serum but not in the skin of mice (not shown).

### BP180/ CXVII-specific antibodies induced subepidermal blistering *in vivo*

Lesional skin of mice was also obtained at the end of the observation period and examined for histological signs of disease by hematoxylin and eosin staining. Subepidermal blister formation accompanied by an inflammatory infiltrate was found in mice receiving either rabbit (Figure 4g) or sheep (Figure 4h) BP180/ CXVII-specific IgG. In contrast, histology from mice injected with control rabbit (not shown) or sheep antibodies (Figure 4i) showed neither inflammatory cells nor dermal epidermal separation. Oesophagus specimens from both diseased or control mice showed no histopathological changes (not shown).

### The extent of skin involvement correlates with levels of serum BP180/ CXVII-specific antibodies in diseased mice

**Figure 5:**
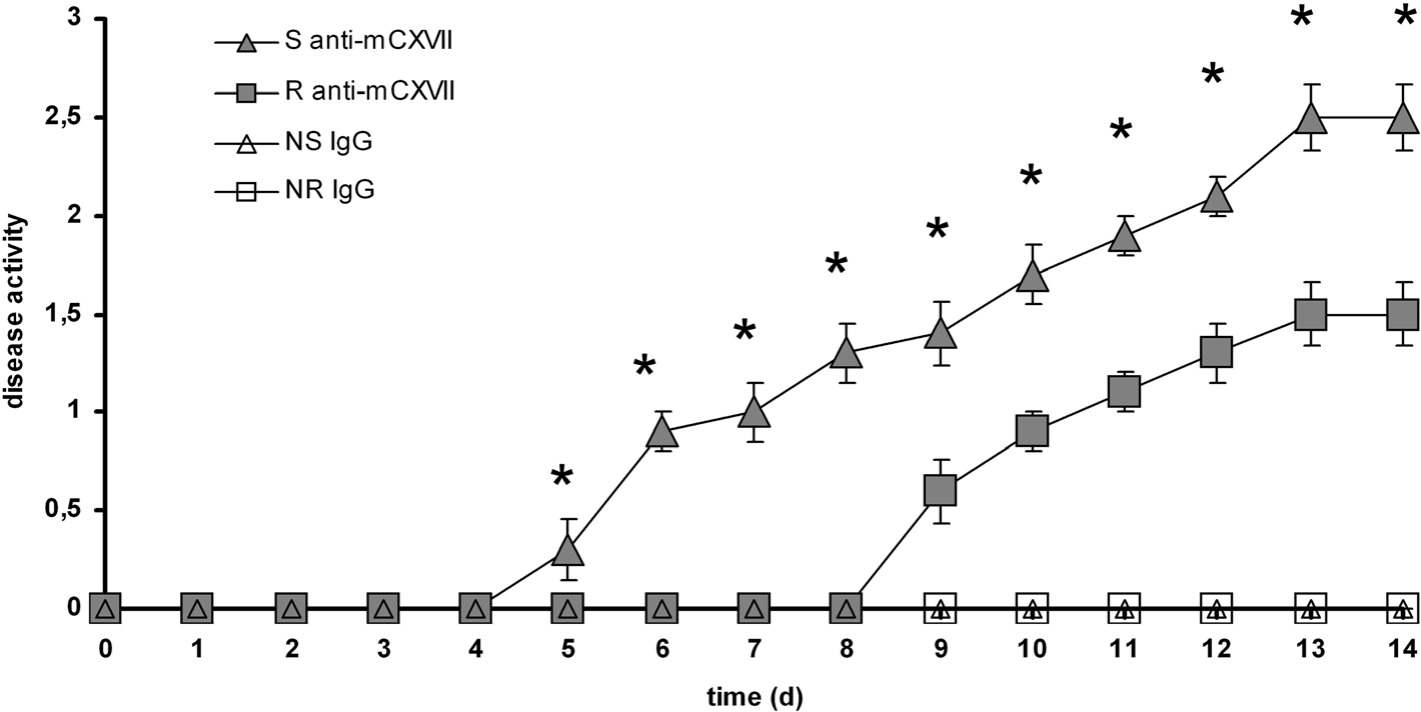
**Anti**-**murine type XVII collagen antibodies of sheep precipitate the onset and potentiate the outcome of disease.** Mice were injected with antibodies to type XVII collagen generated in rabbits (n=10) and sheep (n=10) respectively. After 9–10 days, first lesions appeared in mice transferred with antibodies produced in rabbits. In contrast, mice receiving antibodies generated in sheep presented first lesions after 5–6 days. In addition, at each time-point of the observation period, starting with the day of first lesions appearance, the group injected with pathogenic sheep antibodies had significantly higher disease scores compared to the group injected with pathogenic rabbit anti-mouse collagen BP180/ CXVII antibodies. Groups injected with either control rabbit (n=6) or sheep (n=6) antibodies did not show any clinical lesions throughout the observation period. Means are presented ± s.e.m.; *, significance at p < 0.05 by the chi-square test.

Blood samples were obtained from mice before the first injection (day 0) as well as 7, and 14 days later and assayed by IF microscopy for reactivity to BP180/ CXVII. The results of this analysis are summarized in Table 2. By Spearman’s rank correlation test, titres of serum antibodies strongly correlated with the extent of skin disease both in mice receiving rabbit (r = 0.96) and sheep antibodies respectively (r=0.90). Sheep immune serum showed higher BP180/ CXVII-specific reactivity compared with rabbit immune sera (Table 1). Accordingly, mice receiving BP180/ CXVII-specific sheep IgG showed an earlier onset of disease phenotype and higher scores at any time point when compared to mice injected with the same amount of BP180/ CXVII-specific rabbit IgG whereas mice receiving pre-immune antibodies showed no clinical phenotype (Figure 5).

**Table 2:** Titres of antibodies correlate with the extent of disease in mice

**Group**	**Antibody**^**a**^	**IgG reactivity**^**b**^			**Disease activity**^**c**^		
		**0**^**d**^	**7**	**14**	**0**	**7**	**14**
1	R a-murine BP180/ CXVII	0	128±13	232±30.3	0	0	1.5±0.17
2	NR IgG	0	0	0	0	0	0
3	S a-murine BP180/ CXVII	0	640±149	1248±310	0	1±0.15	2.5±0.17
4	NS IgG	0	0	0	0	0	0

^**a**^ Adult mice were injected every other day with 10mg of either rabbit Ig (group 1, n=10) or sheep IgG (group 3, n=10) against murine BP180/ CXVII. Control groups received similar amounts of control rabbit (group 2, n=6) or sheep (group 4, n=6) antibodies. R a-, rabbit anti-; NR, normal/control rabbit; S a-, sheep anti-; NS, normal/control sheep.

^**b**^ End-point titre of IgG reactivity to the basal membrane was assayed by indirect IF on mouse skin sections.

^**c**^ The extent of skin disease (disease activity) was scored as follows: 0, no lesions; 1, < 10 lesions or < 1% of the skin surface; 2, > 10 lesions or 1-5% of the skin surface; 3, 5-10%; 4, 10-20%; and 5, >20% involvement of the skin surface.

^**d**^ Disease activity and IgG titres are presented at as mean ± s.e.m. at day 0, 7 and 14, respectively. Spearman’s rank correlation test showed significant correlation between antibody titres as detected by IF and disease activity in mice receiving rabbit (group 1) or sheep (group 3) pathogenic antibodies.

## Discussion

Different models for bullous pemphigoid have been generated over the past two decades [14,15,17,19,27]. While the existing models demonstrated the pathogenicity of BP180/ CXVII-specific antibodies, they have several shortcomings. Neonatal mice injected with BP180/ CXVII-specific antibodies do not develop frank skin blistering; epidermal wrinkling and dermal-epidermal separation are induced by applying pressure (rubbing) on the mouse skin. In addition, due to their design, the short observation time of up to three days in the neonatal mice may not be optimal to reproduce the course of chronic disease and for pathogenetic studies and development of new therapeutic approaches. In the present study, to address these drawbacks, we explored whether the passive transfer of BP180/ CXVII-specific antibodies in adult mice results in blistering in the absence of skin rubbing and allows for longer observation times. In a first set of experiments, we generated several fragments of the BP180/ CXVII-specific antibodies, by immunization of rabbits and sheep with the recombinant autoantigen. Since only about 85% of the BP sera react with the immunodominant NC16A domain of BP180/ CXVII by ELISA [38] and most BP sera also recognize other epitopes, we injected, antibodies directed against different fragments of murine BP180 in order to better reflect the broader autoimmune response against BP180 in patients. As shown in figure 2, the polyclonal antibodies generated against recombinant fragments of the murine protein were able to recognize individual fragments by immunoblotting as well as the native form of the protein in cryosections of murine skin activating the complement system, a characteristic feature of several human and experimental autoimmune blistering diseases [15,39-41]. Our present model provides a robust system for further studies dissecting the pathogenic potential of antibodies against different fragments outside the NC16A domain, which may arise in patients as a result of an epitope spreading [42].

To test whether mouse BP180/ CXVII-specific antibodies retain their capacity to recognize the basal membrane and to activate complement *in vivo*, we passively transferred antibodies from immunized animals or control antibodies into adult mice. The antibodies bound at the basement membrane and activated murine complement as shown by direct IF analysis of perilesional biopsies of the skin and oesophagus. In addition, they recruited murine leukocytes and induced skin blistering thus reproducing the human BP at the clinical, immunological and histopathological levels. These findings are in line with similar results of inflammatory blistering in another newly developed passive antibody transfer model of pemphigoid disease in which we pre-immunized adult mice with rabbit IgG followed by injection of collagen XVII-specific rabbit IgG [43]. While having the advantage of an increased inflammatory reaction and more extensive disease in mice compared with the mouse model presented here, the model reported by Oswald *et al.*, has some limitations due to the active immunization with the rabbit IgG, which pose constraints for designing studies addressing the pathophysiology of the pemphigoid disease in patients. Lesions developed at different sites, including the trunk, head, ears and the limbs. Extensive erosions were constantly accompanied by alopecia. Importantly, disease activity in animals receiving pathogenic antibodies correlated with the titres of these antibodies in the sera of injected mice, whereas control mice showed no clinical disease or circulating antibodies. These observations are in line with studies showing a correlation between BP180/ CXVII-specific antibody serum levels and clinical disease in patients [44-46] and their potential to induce tissue damage in an *ex vivo* model for BP [16]. In the present study, in addition to rabbits we have also immunized a sheep in order to test for and compare the outcome of the passive transfer of these antibodies into mice as an alternate source of BP180/ CXVII-specific antibodies. An unexpected result of our present experiments was the fact that sheep antibodies were much more suitable for inducing the disease (Figure 5). This may be due to the higher titre of injected sheep antibodies and/or to a better *in vivo* activation of murine innate immune players, including complement and inflammatory cells. We have obtained relatively high volumes of serum per bleeding and the immune sera showed high titres by indirect IF microscopy on murine skin sections. Since the serum yield of a single sheep can be equivalent to that of eight to ten rabbits, using antibodies generated in sheep may offer a cost-effective alternative for rabbit IgG. A further major advantage of the newly developed mouse model of BP is the fact that it allows for induction of sustained disease and longer observation times of weeks, which can be likely extended to months. This could be advantageous in addressing issues like the specific contribution of different immune players in disease initiation and progression. Probably the most striking difference between the neonatal models and our adult model is represented by the fact that the total IgG amount/g body weight received by adult mice is lower and yet they develop disease without the need to rub their skin. This might be explained at least in part by: 1) the fact that BP is a chronic disease with a complex pathogenesis in which different immune players act in a sequential manner. It is therefore imaginable that limited observation times preclude some immune players to become effectively activated for inducing a frank disease in the neonatal model; hence not only the dose but also the time frame of disease induction may play a critical role; 2) the fact that neonatal mice react differently in terms of activating immune factors *in vivo*. It is worth noting in this context, that neonatal mice injected with antibodies to type VII collagen in the experimental epidermolysis bullosa acquisita mouse model are resistant to blistering despite higher-doses of injected antibodies as compared to adult mice. Susceptibility was rendered in these neonatal mice only after treatment with IL-8 or C5a, two powerful attractants for granulocytes [35].

## Conclusions

We describe here a model of pemphigoid disease in adult mice showing sustained blistering upon injection of BP180/ CXVII-specific antibodies. Pathogenic antibodies for this model may be produced in both sheep and rabbit. Finally, the present model allows for longer observation times. Collectively, these features will greatly facilitate further pathogenetic studies and should prove helpful for translating this information into novel therapeutic strategies for autoantibody-mediated inflammatory diseases.

## Abbreviations

BP: Bullous pemphigoid; BP180/collagen XVII and BP230: BP antigens of 180kDa and 230kDa respectively; DEJ: Dermal-epidermal junction; GST: Gluthatione-S-transferase; IF: Immunofluorescence; IgG: Immunoglobulin G; (NC16)A: 16^th^ non-collagenous domain of BP180/ CXVII.

## Competing interests

The authors declare that they have no competing interests.

## Authors’ contributions

MTC conceived the study, performed experiments and wrote the manuscript. CS conceived and coordinated the study and wrote the manuscript. EL, AGS, AMR, IL, AMC, HS, VPV, ID, AS and OP performed experiments and analysed data. All authors critically read and approved the final manuscript.
